# Guaranteed under the Food and Drug Act!

**Published:** 1912-07-15

**Authors:** J. U. Lloyd


### "GUARANTEED UNDER THE FOOD AND DRUG ACT!"

BY PROF. J. U. LLOYD, M.D.

Physicians, pharmacists, and unfortunately, the people as well, are familiar with the above phrase. It is found on nearly every product manufactured and distributed throughout the United States that can come under the heading of "Foods and Drugs." The effort of those in authority at the time the phrase was formulated, and permission given to use it, was a most worthy one. It was a measure designed to protect the people as well as to serve the interests of the government in behalf of the people. Unquestionably, when it was formulated, but one view of the use to which the phrase could be put, was taken by those in authority, and that view concerned the record of its employment and the serial number attached thereto, which would give the government a hold upon the manufacturer, and would also help protect the dealer distributing the goods. Not for a moment did it ever occur to the authorities that the phrase would be taken advantage of by those who would use it in a manner exactly the opposite the intent of authorities concerned in establishing it.

Within a very short time, however, the original phrase had to be modified, because it was so worded that the consumer accepted that the government actually guaranteed the product so labeled to be all that the manufacturer claimed it to be. In this direction, we recall the manner in which foods and drugs and medicines were advertised as being fathered by the United States' government, both as concerns their origin and their qualities.

To such an extent was this imposition carried, as very soon to necessitate a change in the wording of the phrase, the object being to teach the people that the Government *did not guarantee anything* concerning the quality of the material under the label. But, alas, the new phrasing also carries a self-evident misleading opportunity, as demonstrated by the facts of experience. The people, with child-like simplicity, yet accept that the phrase "Guaranteed under the Food and Drug Act," is a Government commendation of the preparation, and a Government guarantee. Alas, too well we know that, as editorially expressed by the *Journal of the American Medical Association*, "The most pernicious humbug is 'guaranteed' in this way."

Foreseeing that all these impositions would arise, and that the term "guaranteed" or "liscensed" or "allowed," or whatever else might be used by the Government, would be seized upon by those intent on establishing wrong, or of getting an advertisement under the wing of the Government, we at the outset protested against any phrase of that description being allowed anyone whomsoever. We contended that it was unnecessary, because the Government could easily find the manufacturer of any product that crossed a state line if it was deemed necessary so to so, and could find the maker of any product that emanated from any laboratory or from any manufacturer of drugs or foods, when that product entered commerce. We believe that if any phrase or serial number be awarded the reputable manufacturer, it would be at once taken advantage of by others, and used unfairly, with all the energy at their command. This argument was made before the first "Guaranteed" form was accepted. It was repeated even more strenuously when the attempt was made to revise that form. It seems to us now that the experience of the past must be sufficient to illustrate the need of the immediate abondonment of the whole process. It seems to us now as though the Government can and should immediately require that all reference to the Government's "guarantee" should, at a date fixed in the future, be thereafter excluded from every print accompanying every food or drug product. If there be a necessity for any record in Washington, (and this in our opinion, is not necessary), let it be simply the one statement, "Serial Number——," without any reference whatever to "Guarantee," or to the Government, or to any authority whatsoever.

It may be urged that the leaving off from the label the phrase "Guaranteed Under the Food and Drugs Act," would now subject a manufacturer dropping it from his label, to suspicion and perhaps lead his patrons, through a misapprehension, to purchase from his competitors. It might also be claimed that label changes would be expensive. In reply to the first argument we will say that one manufacturer of remedial agents, foreseeing the above complication from the beginning, decided not to place the phrase on any product emanating from his laboratory, and he knows of no invidious distinction or any complaint having been made, by anyone whomsoever. To the second we will say, men in business expect continuous expenses, and as a rule are willing to help in the betterment of conditions.
—*Eclectic Medical Journal.*

